# BDNF Overexpression Enhances Neuronal Activity and Axonal Growth in Human iPSC-Derived Neural Cultures

**DOI:** 10.3390/ijms26157262

**Published:** 2025-07-27

**Authors:** Alba Ortega-Gasco, Francesca Percopo, Ares Font-Guixe, Santiago Ramos-Bartolome, Andrea Cami-Bonet, Marc Magem-Planas, Marc Fabrellas-Monsech, Emma Esquirol-Albala, Luna Goulet, Sergi Fornos-Zapater, Ainhoa Arcas-Marquez, Anna-Christina Haeb, Claudia Gomez-Bravo, Clelia Introna, Josep M. Canals, Daniel Tornero

**Affiliations:** 1 Laboratory of Neural Stem Cells and Brain Damage, Department of Biomedical Sciences, Institute of Neurosciences, University of Barcelona, 08036 Barcelona, Spain; francesca.percopo@edu.unifi.it (F.P.); afontgui100@alumnes.ub.edu (A.F.-G.); bramossa23@alumnes.ub.edu (S.R.-B.); acamibon30@alumnes.ub.edu (A.C.-B.); mmagempl7@alumnes.ub.edu (M.M.-P.); mfabremo24@alumnes.ub.edu (M.F.-M.); eesquial7@alumnes.ub.edu (E.E.-A.); lgoulego45@alumnes.ub.edu (L.G.); sfornoza13@alumnes.ub.edu (S.F.-Z.); ainhoaa2698@gmail.com (A.A.-M.); anna.haeb@ub.edu (A.-C.H.); claudia.gomez-bravo@inserm.fr (C.G.-B.); 2 Institut d’Investigacions Biomèdiques August Pi i Sunyer (IDIBAPS), 08036 Barcelona, Spain; jmcanals@ub.edu; 3 Laboratory of Stem Cells and Regenerative Medicine, Department of Biomedical Sciences, Faculty of Medicine and Health Sciences, Institute of Neurosciences, and Creatio, Production and Validation Center of Advanced Therapies, University of Barcelona, 08036 Barcelona, Spain; cleliaintrona@msn.com; 4 Centro de Investigación Biomédica en Red sobre Enfermedades Neurodegenerativas (CIBERNED), 28029 Madrid, Spain

**Keywords:** brain-derived neurotrophic factor (BDNF), neural progenitor cells (NPCs), human iPSCs, axonal growth, neuronal activity, regenerative medicine

## Abstract

As the global population continues to age, the incidence of neurodegenerative diseases and neural injuries is increasing, presenting major challenges for healthcare systems. Due to the brain’s limited regenerative capacity, there is an urgent need for strategies that promote neuronal repair and functional integration. Brain-derived neurotrophic factor (BDNF) is a key regulator of synaptic plasticity and neuronal development. In this study, we investigated whether constitutive BDNF expression in human induced pluripotent stem cell (iPSC)-derived neural progenitor cells (NPCs) enhances their neurogenic and integrative potential in vitro. We found that NPCs engineered to overexpress BDNF produced neuronal cultures with increased numbers of mature and spontaneously active neurons, without altering the overall structure or organization of functional networks. Furthermore, BDNF-expressing neurons exhibited significantly greater axonal outgrowth, including directed axon extension in a compartmentalized microfluidic system, suggesting a chemoattractive effect of localized BDNF secretion. These effects were comparable to those observed with the early supplementation of recombinant BDNF. Our results demonstrate that sustained BDNF expression enhances neuronal maturation and axonal projection without disrupting network integrity. These findings support the use of BDNF not only as a therapeutic agent to improve cell therapy outcomes but also as a tool to accelerate the development of functional neural networks in vitro.

## 1. Introduction

Neurological disorders, including neurodegenerative diseases and traumatic brain injuries, present a significant challenge to global health [1]. These conditions are characterized by neuronal loss and dysfunction, leading to cognitive and behavioral declines in affected individuals [2]. However, in many cases, the human brain is unable to fully recover, highlighting the need for new and effective neuroregenerative treatments.

In this context, stem cell-based therapies are actively being developed for their potential to restore neuronal function [3] and enable true cell replacement in neurodegenerative and traumatic brain conditions in the Central Nervous System [4]. A wide range of stem cell strategies are under investigation, with unique advantages such as multipotency, accessibility, and regenerative capacity, and specific limitations related to ethical concerns, variability in therapeutic efficacy, and especially integration into host neural circuits. Preclinical studies and early-phase clinical trials have demonstrated encouraging results, including improvements in cognitive and motor function. One of the most advanced applications of stem cell-based neuronal replacement is in the treatment of Parkinson’s disease (PD) in which human pluripotent stem cell-derived dopaminergic progenitors are currently being evaluated in Phase I/II clinical trials across multiple countries, including Sweden, Japan, and the United States [5,6,7,8].

However, significant limitations continue to hinder widespread clinical translation. Key challenges include determining the most suitable cell type, optimizing delivery routes, transplantation timing, dosing, and ensuring long-term safety and efficacy. Immune rejection remains a major concern, particularly for allogeneic transplants, prompting the development of HLA-matched donor cell banks and genome-edited “universal donor” cell lines, alongside continued reliance on immunosuppression protocols [9,10]. Another critical hurdle is ensuring the functional integration of transplanted cells into host neural networks, including accurate synaptic connectivity and regulated neurotransmitter release. To address these issues, next-generation stem cell strategies are focusing on enhanced safety and functional performance through non-viral reprogramming methods, refined cell-sorting protocols, and bioengineering approaches, such as the incorporation of biomaterials or gene-modified grafts capable of delivering neurotrophic factors like BDNF or GDNF [11,12,13,14].

BDNF is a well-characterized neurotrophin that is expressed during the development and maintenance of the Central Nervous System [15,16]. It plays numerous crucial roles in neuronal survival, synaptic plasticity, neurotransmitter regulation, and network remodeling [16,17]. But BDNF is also recognized for its role in many different neurodegenerative and psychiatric disorders [18,19]. Because of its broad neuroprotective effects, BDNF has long been considered a therapeutic target for neurological disorders. To address the therapeutic benefits of the introduction of BDNF, different delivering strategies have been explored, including recombinant BDNF peptides, mRNAs, polymer-based sustained release systems, or gene-editing platforms (AAV vectors, CRISPR-Cas9) [20]. However, they face several important limitations, including limited capacity for structural repair and, in some cases, challenges in efficient delivery and restricted spatial and temporal control. Additionally, their effectiveness often depends on the presence of residual neural networks and can vary significantly between patients. Therefore, these approaches are valuable for protecting existing neurons and enhancing neural plasticity, but they are insufficient for replacing lost neurons or fully restoring complex neural circuits in extensively damaged regions of the Central Nervous System. Combining non-cellular strategies with cell-based therapies may help overcome some of these challenges. Indeed, recent advances demonstrate that integrated gene and cell-based delivery approaches hold promise for addressing these limitations more effectively. For instance, the stem cell-based delivery of BDNF using genetically engineered stem or progenitor cells has demonstrated robust neuroregenerative potential in vivo [12,21,22,23]. In a spinal cord transection model, human bone-marrow-derived mesenchymal stem cells (MSCs) overexpressing BDNF significantly promoted corticospinal and serotonergic axonal sprouting, enhanced motor neuron survival, and improved locomotor function compared to unmodified MSCs [23]. MSCs genetically modified to overexpress BDNF significantly improve functional recovery and reduce brain damage in a stroke model. These MSC-BDNF cells enhance neuroprotection by reducing neuronal apoptosis and promoting tissue repair. This approach shows promise as a potential therapy for ischemic stroke [12,24].

The use of NPCs or neural stem cells (NSCs) has also started to be explored. In Alzheimer’s disease mouse models, primary cultures of mouse NSCs engineered to overexpress BDNF showed improved survival, neuronal differentiation, synaptic maturation, and cognitive performance; these effects were reversed by BDNF knockdown, confirming its mechanistic relevance [21]. In conclusion, stem cells engineered to express BDNF represent a clinically relevant approach to enhancing the regenerative capacity of cell therapies for CNS injuries and disorders. Both MSCs and NSCs modified to deliver BDNF have outperformed unmodified cells in promoting neuronal survival and axonal regeneration. However, additional work is required to optimize their efficacy, safety, and scalability for clinical applications.

In the present work, we aimed to test, in vitro, whether constitutive BDNF expression in human iPSC-derived NPCs could enhance the efficiency of cell transplants by promoting survival and improving the integration capabilities of the graft.

## 2. Results

### 2.1. BDNF Can Be Overexpressed in NPCs with a Fluorescent Calcium Indicator

**Figure 1:**
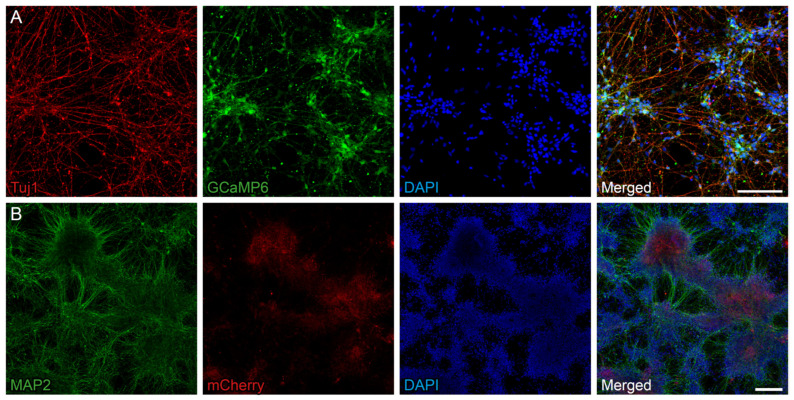
Overexpression of BDNF in human iPSC-derived NPCs expressing a calcium indicator. (**A**) Immunofluorescence staining of differentiating lt-NES at DD25 cells shows βIII-tubulin (red) and GCaMP6s (green), confirming early neuronal identity and expression of the calcium indicator. Nuclei are counterstained with DAPI (blue). (**B**) Immunofluorescence staining for MAP2 (green) and mCherry (red) indicates successful BDNF overexpression in lt-NES cells at DD35 without impairing neuronal maturation, as evidenced by MAP2 positivity. Nuclei are counterstained with DAPI (blue). Scale bar in A = 100 µm and in B = 200 µm.

In this study we used human iPSC-derived long-term neuroepithelial-like stem (lt-NES) cells as a source for NPCs. Upon transplantation, these cells have shown the capacity to survive, generate mature cortical neurons, and functionally integrate into the stroke-damaged rat cortex [25,26]. We first transformed this cell to incorporate the genetically-encoded calcium indicator GCaMP6s, to monitor neuronal activity across the differentiation process. Upon growth factor withdrawal, lt-NES cells gradually lose stemness properties and generate in vitro mature neurons, astrocytes, and oligodendrocytes [27,28]. Since GCaMP6s expression was driven by the human SynI promoter, derived neurons at differentiation day 25 (DD25), identified by the expression of βIII-tubulin, also express the calcium indicator (Figure 1A).

A lentiviral vector was used to overexpress the growth factor BDNF in this system. Successful BDNF expression was confirmed via immunohistochemistry (Figure 1B) at DD35 and quantitative PCR (Figure S1). Importantly, BDNF overexpression did not impair the ability of lt-NES cells to differentiate into mature neurons, as indicated by MAP2 immunoreactivity (Figure 1B).

### 2.2. BDNF Overexpression Enhances Neuronal Differentiation of Human iPSC-Derived NPCs

**Figure 2:**
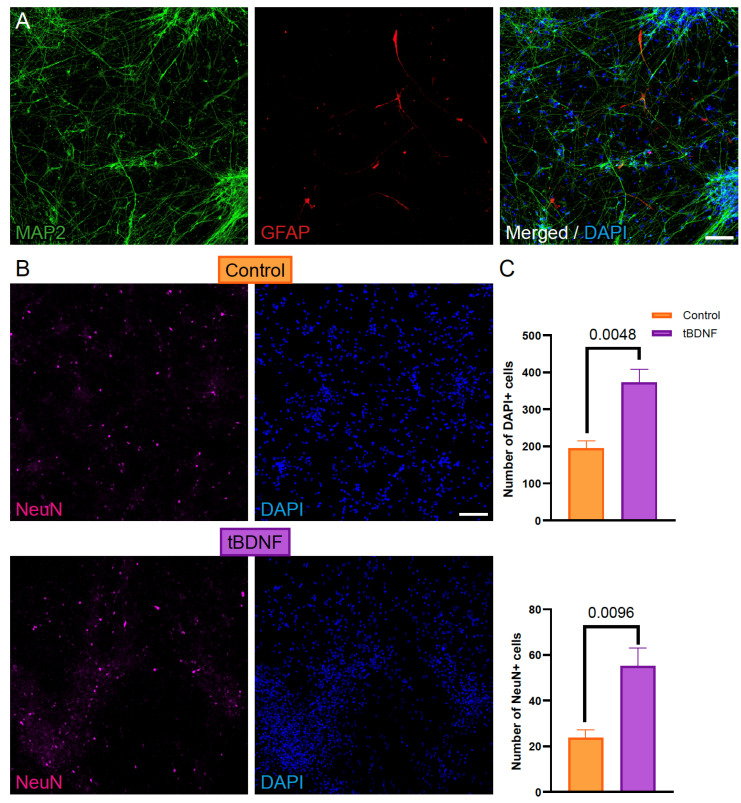
BDNF overexpression enhances neuronal differentiation of human iPSC-derived NPCs. (**A**) Representative image of immunofluorescence staining of control NPCs at DD56 into mature neurons (MAP2-positive, green) and astrocytes (GFAP-positive, red). Nuclei are counterstained with DAPI (blue) in merged image. (**B**) Immunostaining for NeuN (purple) and DAPI (blue) reveals an increased number of NeuN-positive cells in tBDNF cultures compared to controls. (**C**) Quantification of DAPI-positive (**top**) and NeuN-positive (**bottom**) cells confirms enhanced cell viability or proliferation and neuronal differentiation in the tBDNF condition (*n* = 4). Data are presented as total cell counts per field of view on each condition. *p*-values are depicted in each graph. Scale bar in A = 100 µm and B = 50 µm.

To evaluate the effect of BDNF overexpression on neuronal differentiation, we generated cultures from human iPSC-derived NPCs genetically modified to express BDNF (tBDNF) and compared them with unmodified control NPCs. Immunostaining on neural cultures at DD56 revealed a significant increase in the number of NeuN-positive cells in tBDNF cultures compared to controls (Figure 2A,B), indicating enhanced neuronal differentiation. Additionally, total cell counts (DAPI-positive nuclei) were higher in cultures from the tBDNF condition (Figure 2C), suggesting improved cell viability or proliferation associated with this effect.

### 2.3. BDNF Overexpression Increases the Number of Active Neurons Without Altering Network Topology

**Figure 3:**
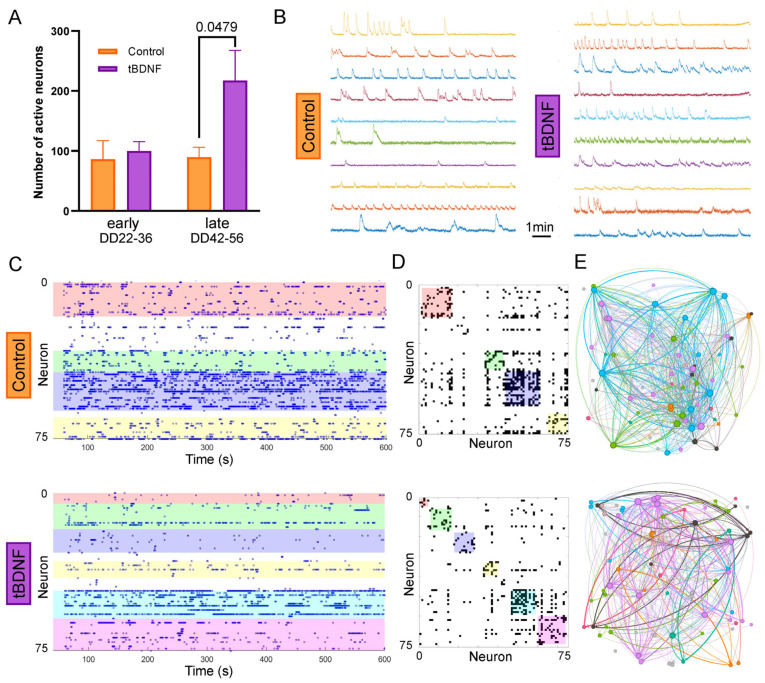
BDNF overexpression increases the number of active neurons without altering network topology. (**A**) Quantification of spontaneously active neurons per field of view reveals a significant increase in the tBDNF condition compared to the control (for early timepoint *n* = 4, for late timepoint *n* = 5). A *p*-value is depicted when >0.05 (**B**) Representative intracellular calcium traces illustrate spontaneous activity in control (**left**) and tBDNF (**right**) cultures at DD56. (**C**) Raster plots of neuronal activity show clusters (color boxes) of temporally coactive neurons (functional communities) in control (**top**) and tBDNF (**bottom**) cultures at DD56 (*y*-axis depicts normalized fluorescence intensity in arbitrary units). (**D**) Connectivity matrices computed from the raster plots through transfer entropy, illustrating the significant exchange of information among neurons and the detected community structure. (**E**) Graph-based connectivity maps demonstrate similar overall network organization between control (**top**) and tBDNF (**bottom**) cultures at DD56 (the size of each point is proportional to the connectivity).

To assess the functional properties of the differentiated neuronal cultures, we performed calcium imaging to quantify spontaneous neuronal activity from DD22 to DD56. For the later timepoint, cultures derived from NPCs overexpressing BDNF (tBDNF) exhibited a higher number of active neurons per field of view compared to control cultures (Figure 3A), which is consistent with the increase in NeuN+ cell counts observed previously.

To determine whether BDNF overexpression also affected the organization of the resulting neuronal networks, we performed graph-based analyses of the calcium imaging data. Graph metrics such as global efficiency, modularity, and average number of connections per neuron [29,30] did not differ significantly between the tBDNF and control neural networks (Figure S2). Representative raster plots and activity traces showed similar patterns of spontaneous events across all conditions (Figure 3B–E), indicating that, while BDNF increases neuronal differentiation and the number of functionally active neurons, it does not alter the overall topology of network connectivity in these cultures.

### 2.4. BDNF Promotes Directional Axonal Outgrowth in a Microfluidic System

**Figure 4:**
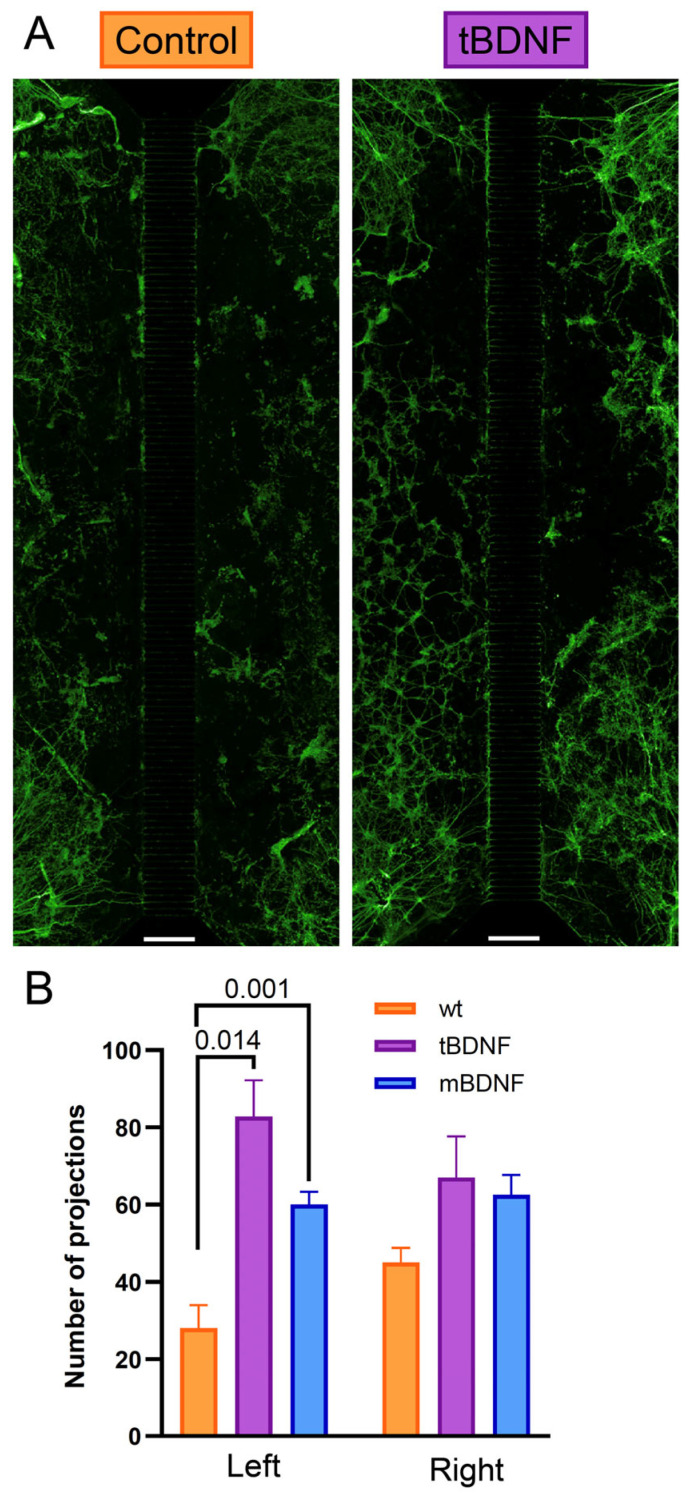
BDNF promotes directional axonal outgrowth in a microfluidic system. (**A**) Representative immunofluorescence images of microfluidic chips stained for MAP2 (green) at DD52 show axonal projections extending through microchannels (400 µm) between the two compartments in control (**left**) and tBDNF (**right**) conditions. In the latest, tBDNF cells are located in the right compartment. (**B**) Quantification of axonal projections entering the microchannels from each side in control, tBDNF, and mBDNF-treated conditions (*n* = 4 for each condition). A *p*-value is depicted for each comparison when >0.05. Scale bar in A = 400 µm.

To investigate whether BDNF expression can influence axonal guidance in addition to promoting outgrowth, we employed a microfluidic chip system (Xona Microfluidics) consisting of two separate compartments connected by microchannels 400 µm in length. NPC-derived neurons were seeded in both compartments. In the experimental condition, cells overexpressing BDNF (tBDNF) were plated in one compartment, and control cells were seeded in the opposite one. Strikingly, at DD30, we observed a significant increase in axonal projections entering the channels originated from the opposite compartment to the one containing BDNF-expressing cells (Figure 4A). This suggests that BDNF acts as a chemoattractant, promoting directional axonal growth across the microchannels.

As a positive control, we supplemented recombinant BDNF to the culture medium (mBDNF) in one of the compartments with control-derived cells and observed a similar enhancement of axonal projection density as the one observed in the tBDNF condition (Figure 4B). Thus, the directional bias observed with localized tBDNF expression indicates that cell-intrinsic BDNF not only enhances axon growth but may also create local gradients sufficient to influence axonal targeting in vitro.

## 3. Discussion

Our experiments demonstrate that human iPSC-derived NPCs differentiated into cortical phenotype can be used to ectopically express BDNF and secrete it, enhancing neuronal activity and axonal outgrowth, features that are important for neuroregeneration. Moreover, this effect did not interfere with the capacity of the cells to generate functional neural networks in vitro. These findings support the concept that the localized, continuous delivery of BDNF via genetically modified neural progenitor cells (NPCs) may represent a viable strategy to enhance the efficacy of cell therapies for neurodegenerative disorders.

We first confirmed robust BDNF overexpression in the engineered tBDNF line, observing the presence of the transcript via quantitative PCR and the reporter (mCherry) via immunohistochemistry. Despite this overexpression, the proportion of NeuN-positive mature neurons did not differ between tBDNF and control cultures; however, an increase in the total number of mature neurons was observed. This increase was accompanied by a higher total cell count, suggesting that BDNF overexpression may enhance overall cell numbers. While this effect could theoretically result from either increased proliferation or reduced cell death, we consider the former more likely, given the nature of BDNF as a growth-promoting factor and the lack of obvious differences in cell death when assessed via brightfield microscopy. Future studies employing specific markers of proliferation (e.g., Ki67, BrdU) or apoptosis (e.g., cleaved caspase-3) will be necessary to clarify the underlying mechanisms driving this increase in cell number.

At the later stages of differentiation, calcium imaging revealed that tBDNF cultures contained nearly twice as many spontaneously active neurons as wild-type controls. This increase in neuronal activity is consistent with the known ability of BDNF to support synaptic plasticity and excitability [31]. However, graph-theoretical analysis of the activity patterns showed no significant differences in neuronal network descriptors. These results indicate that while BDNF promotes functional maturation at the cellular level, it does not disrupt the overall architecture or organization of the neuronal network, thereby avoiding the risk of hyperconnectivity or disorganized circuit formation.

Additionally, experiments using a microfluidic chamber system showed a marked increase in axonal projections in the tBDNF condition relative to controls. Notably, axons extended preferentially from the opposite compartment to the one containing BDNF-expressing cells, suggesting a chemoattractive effect of sustained endogenous BDNF secretion, as it was already observed [32]. This directional bias was similarly observed in cultures treated with recombinant BDNF (mBDNF) in one of the compartments with control cells. These findings imply that localized, continuous BDNF expression can generate concentration gradients capable of guiding axonal projections, a mechanism with potential relevance for improving anatomical integration in cell replacement strategies. To confirm that this effect is specifically mediated by BDNF secretion from the overexpressing neurons, future experiments could include the application of TrkB receptor bodies to both compartments to scavenge extracellular BDNF. This would allow us to determine whether blocking BDNF signaling abolishes the observed directional axonal growth, thereby providing more direct evidence that BDNF release is the primary driver of this effect.

Several limitations of this study should be acknowledged. The duration of the differentiation protocol may not be sufficient to fully reveal the long-term effects of BDNF on neuronal subtype specification and synaptic remodeling. Extending the culture period could uncover additional effects on network maturation and stability. Furthermore, in vivo validation will be essential to determine whether the improvements observed in vitro can translate into enhanced graft integration, survival, and functional recovery in models of neurodegeneration or brain injury. This will involve the use of cutting-edge technologies, such as calcium imaging, optogenetics, and monosynaptic tracing [33] with the aim of exploring true cell replacement [4,34]. These future experiments will also help clarify the contribution of BDNF-expressing grafts to host circuitry reestablishment and behavioral restoration.

In conclusion, the present findings highlight the dual potential of BDNF as both a therapeutic agent and a research tool. In the therapeutic context, BDNF may support neuronal survival, maturation, and connectivity following transplantation. In parallel, it can serve as a valuable modulator for accelerating and refining the development of functional neuronal networks in vitro, which has important implications for disease modeling, drug screening, and neuroengineering applications.

## 4. Materials and Methods

### 4.1. Cell Line and Cortical Priming

Human iPSC-derived lt-NES cells were produced from skin fibroblasts as previously described [35]. The cells were maintained in a proliferative state in the presence of growth factors (FGF and EGF) and B27 (Gibco, Waltham, MA, USA). For differentiation, lt-NES cells were primed towards a cortical neuronal phenotype according to the stablished protocol [28] with minor changes. Briefly, growth factors and B27 were omitted, and cells were cultured at low density in differentiation-defined medium in the presence of BMP4 (10 ng/mL, R&D Systems, Minneapolis, MN, USA), Wnt3A (10 ng/mL, R&D Systems) and cyclopamine (1 mM, Calbiochem, San Diego, CA, USA) for 5 days. Then, neuronal progenitors were transferred to their final destination after coating with poly-L-ornithne (Sigma-Aldrich, St. Louis, MO, USA) and hr-Laminin 521 (Biolamina, Sundbyberg, Sweden) and left to mature in BrainPhys media (Stem Cell Technologies, Vancouver, BC, Canada) supplemented with B27 (without retinoic acid, Gibco).

### 4.2. Lentiviral Transfection

Proliferative lt-NES cells were infected with the lentivirus (0.5 µL/mL from a titer of 2·10^8^ TU/mL) containing the plasmid for the expression of the fluorescent calcium indicator GCaMP6s under control of the Syn-I promoter including a puromycin selection cassette (Vector Builder, Chicago, IL, USA). Two days later, puromycin (1 µg/mL, Gibco) was added to the media for selection.

For the overexpression of BDNF, the resulting line of lt-NES cells, in a proliferative state, was infected with a lentiviral vector (0.5 µL/mL from a titer of 4.1·10^8^ TU/mL) carrying an EF1a-BDNF-mCherry construct (Vector Builder) for the constitutive expression of BDNF, together with cytosolic mCherry as a reporter. The information about the BDNF sequence used in the design can be found in Supplemental Information.

### 4.3. Quantitative PCR

Quantitative PCR was performed on DNA extracted from lt-NES cells (Maxwell^®^ 16 Instrument, Promega, Madison, WI, USA) to confirm the incorporation of GCaMP6s and BDNF into the genome. The concentration of the genomic DNA samples was measured using a Nanodrop Spectrophotometer (NanoDrop Technologies, Wilmington, DE, USA). The PCR reactions were prepared using 100 ng of DNA for each sample. GCaMP6s and BDNF were analyzed as targeted genes using two pairs of forward and reverse primers previously designed, and 18-RNA and β-actin were used as housekeeping genes.

### 4.4. Immunocytochemistry

Cells at different timepoints, depending on the experiment, were fixed with paraformaldehyde 4% for 15 min at room temperature. After fixation, cells were incubated in blocking solution, containing PBS, 0.03% Triton X-100 (Sigma-Aldrich, and 5% NDS (Jackson Immunoresearch, West Grove, PA, USA), for 45 min at room temperature. Primary antibodies (Table S1) were applied, diluted in blocking solution, and incubated overnight at 4 °C. Hereafter, corresponding fluo-labeled secondary antibodies were incubated for 90 min at room temperature. Finally, cells were counterstained with DAPI (ThermoFisher Scientific, Waltham, MA, USA). Images were acquired using a confocal microscope ZEISS LSM 880 (Carl Zeiss AG, Oberkochen, Germany) and Opera Phenix Plus High-Content Screening System (Revity, PerkinElmer, Waltham, MA, USA).

### 4.5. Quantifications

For cell counts, at least 4 fields of view (20× objective) in confocal maximum projection images from 3 different cultures were analyzed to identify total DAPI-positive nuclei and NeuN-positive nuclei using Cell Profiler 4.2.8. software tools.

For projection counts, 4 identical confocal maximum projection tile images from 2 cultures of each condition and the side of the chip were used (total n = 4, per condition). Tile images were generated with 12 (2 × 6) pictures taken with a ×10 objective and an overlap of 10% for the stitching. Quantification was performed manually using the ImageJ 1.54g software multi-point tool.

### 4.6. Calcium Fluorescence Imaging

Intracellular calcium fluorescence imaging using GCaMP6s was used to monitor dynamic changes in neuronal activity. Culture dishes contained a grid that allows recording the same region of the plate at different timepoints. Calcium recordings were conducted on an Olympus IX70 inverted epifluorescence microscope (Olympus Corporation, Tokyo, Japan) equipped with a high-speed camera (Hamamatsu Orca Flash 4.0, Hamamatsu Photonics, Hamamatsu, Japan) and a LED-light source of a 488 nm wavelength for GCaMP6s fluorescence recordings. Spontaneous neuronal activity was evaluated every 3–4 days for 10 min per dish, and cultures were tracked from around DD22 for approximately 4 weeks, covering from the onset of GCaMP6s expression to stable overall activity. Each of the three co-culture repetitions per cell line was tracked with a total of six recordings per culture dish for both independent differentiations, to monitor the changes in neuronal activity along culture maturation. Images were acquired with the imaging software Hokawo 2.10 at 20 frames per second (fps), in 8-bit greyscale format. Cells were visualized with an ×10 objective lens, providing a field of view of 1.1 × 1.1 mm^2^ and a spatial resolution of 1.08 μm/pixel.

### 4.7. Calcium Imaging Analysis

Recorded movies of intracellular calcium fluorescence were analyzed with NETCAL 7.4.1 software (http://www.itsnetcal.com/ (accessed on 26 July 2025)). For each experiment, high-contrast fluorescence images of the average recording were generated. Neuronal somas were identified automatically and refined manually in the resulting images and assigned as regions of interest (ROIs), and the average fluorescence (grayscale level) within each ROI along the recording was then extracted, providing the raw fluorescence traces for each neuron. These individual fluorescence traces were then normalized considering the fluorescence value of each neuron at rest. Noise in fluorescence traces was reduced by using a moving average filter, where each data point was replaced by the average of a 5-point window (the point itself and its two immediate neighbors on either side). Additionally, a high-pass filter preserving frequencies above 0.01 Hz was applied to eliminate slow fluorescence fluctuations (on the order of minutes) and those that were unrelated to neuronal activity events. Neuronal activations (spikes) were inferred using the Oasis method, which determines the number of neuronal activity events based on the amplitude and shape of the fluorescence signal. The inferred spikes, corresponding to neuronal spontaneous activity, were then visualized in raster plots.

Then, network activity was computed as the percentage of neurons in the network that had at least 2 inferred activations along the recording relative to the total number of neurons recorded, whereas the mean activity was defined as the number of spikes per neuron per minute. The population activity was computed from the raster plots and showed the tendency of neurons in the network to activate collectively in a short time window. The height of the peaks in the population activity was termed the ‘collective event size’ and varied between 0 (no activity) and 1 (network-wide activation). This descriptor was used to quantify the degree of coordinated activity in the network, termed the ‘synchronization level’, which is defined as the percentage of the collective event size comprising at least 25% of neurons within the network relative to the total number of collective events along the recording.

To infer statistical relationships among the activity patterns of neurons, functional connectivity was computed on the inferred spike trains using generalized transfer entropy (GTE) as previously described [29] and was run in MATLAB 2021a. This measurement provided adjacency connectivity matrices that captured the significant exchange of information among neurons, which were then visualized as spatial connectivity maps with Gephi 0.10.1. Computed matrices were used to infer network properties and compare different culture conditions.

Adjacency connectivity matrices were analyzed using the “Brain Connectivity Toolbox” [36], to quantify the topological organization in the cultures. Three main network properties were used: global efficiency (G_E_), modularity (Q), and average connectivity 〈k〉. G_E_ describes the capacity of neurons to exchange information throughout the whole network. It varies between 0 (neurons disconnected) and 1 (all neurons reachable through just one connection). Higher G_E_ values reflect highly integrated networks, whereas lower values indicate strong segregation. Q refers to the tendency of neurons to form functional communities, defined as groups of neurons that are more connected within their groups than with neurons in other groups, and ranges between 0 (the entire network shaped as a unique community) and 1 (each neuron is an isolated community). Values around 0.3 or higher indicate the existence of clear communities. Finally, 〈k〉 refers to the average number of connections per neuron, obtained by computing the number of links of a neuron with all the others and then dividing by the number of neurons.

### 4.8. Statistical Analysis

Statistical analysis was performed with GraphPad Prism 10 software. The normality of data distribution was assessed using the Shapiro–Wilk test. Two-way ANOVA was used to compare control and BDNF groups across different timepoints, while an unpaired t-test was used to compare specific stages when significant differences were observed. When needed for multiple comparisons, we applied the Bonferroni correction. Significance was set at *p* < 0.05. Data were averaged from a minimum of four values per condition. Results are plotted as the mean ± SEM.

## Data Availability

No database was created. The original contributions presented in this study are included in the article and Supplementary Materials. Further inquiries can be directed to the corresponding author(s).

## Supplementary Materials

The supporting information can be downloaded at: https://www.mdpi.com/article/10.3390/ijms26157262/s1.

## Abbreviations

The following abbreviations are used in this manuscript:

BDNF	Brain-derived neurotrophic factor
NPCs	Neural progenitor cells
iPSC	Induced pluripotent stem cells
lt-NES	Long-term neuroepithelial-like stem
NSCs	Neural stem cells
MSCs	Mesenchymal stem cells
