# Supplementary material for: BDNF Overexpression Enhances Neuronal Activity and Axonal Growth in Human iPSC-Derived Neural Cultures
Source: Int J Mol Sci. 2025 Jul 27;26(15):7262. doi: 10.3390/ijms26157262 (PMC12346939; doi:10.3390/ijms26157262)
Supplement: Supplementary file 1 [file ijms-26-07262-s001.zip › Figure S1.pdf]

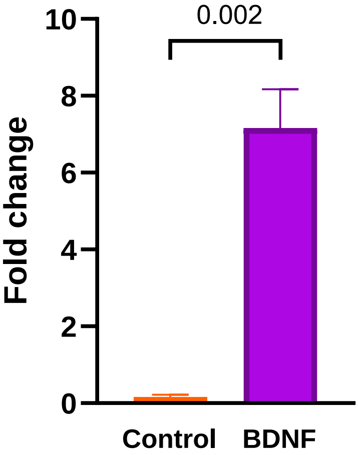

Figure S1: RT-qPCR data revealed expression of BDNF in the NPCs after infection as compared with control non-infected cells. Average from triplicate meassurement is shown using 18-RNA as housekeeping gene and error bars represent standard error of mean. p-value is depicted in the graph.
