# Supplementary material for: BDNF Overexpression Enhances Neuronal Activity and Axonal Growth in Human iPSC-Derived Neural Cultures
Source: Int J Mol Sci. 2025 Jul 27;26(15):7262. doi: 10.3390/ijms26157262 (PMC12346939; doi:10.3390/ijms26157262)
Supplement: Supplementary file 1 [file ijms-26-07262-s001.zip › Figure S2.pdf]

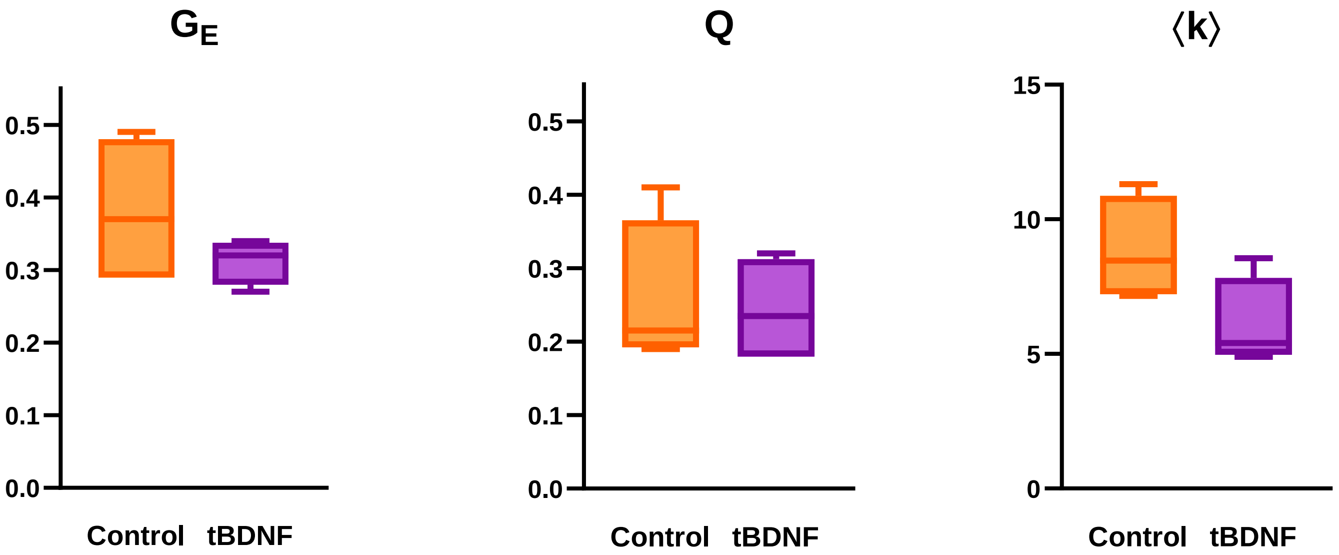

Figure S2. Graphs summarizing the network analysis showing global efficiency (G_E), modularity (G) and average connectivity **⟨k⟩** in control and tBDNF conditions.
