# Supplementary material for: BDNF Overexpression Enhances Neuronal Activity and Axonal Growth in Human iPSC-Derived Neural Cultures
Source: Int J Mol Sci. 2025 Jul 27;26(15):7262. doi: 10.3390/ijms26157262 (PMC12346939; doi:10.3390/ijms26157262)
Supplement: Supplementary file 1 [file ijms-26-07262-s001.zip › Supplementary Information.pdf]

Antibody	Specie	Code	Company
MAP2	Chicken	ab92434	Abcam
GFP	Goat	ab6662	Abcam
βIII-tubilin	Mouse	T8660	Sigma
RFP	Mouse	390 011	Synaptic Systems
GFAP	Mouse	C9205	Sigma
NeuN	Rabbit	24307	Cell Signaling

Table S1. List of primary antibodies used in the study.

Supplemental information: sequence of the hBDNF in the construct.

ATGTGTGGAGCCACCAGTTTTCTCCATGAGTGCACAAGGTTAATCCTTGTTACTACTCAGAAT
GCTGAGTTTCTACAGAAAGGGTTGCAGGTCCACACATGTTTTGGCGTCTACCCACACGCTTCT
GTATGGCATGACTGTGCATCCCAGAAGAAGGGCTGTGCTGTGTACCTCCACGTTTCAGTGGA
ATTTAACAAACTGATCCCTGAAAATGGTTTCATAAAGTTCCACCAGGTGAGAAGAGTGATGAC
CATCCTTTTCCTTACTATGGTTATTTCATACTTTGGTTGCATGAAGGCTGCCCCCATGAAAGAA
GCAAACATCCGAGGACAAGGTGGCTTGGCCTACCCAGGTGTGCGGACCCATGGGACTCTGG
AGAGCGTGAATGGGCCCAAGGCAGGTTCAAGAGGCTTGACATCATTGGCTGACACTTTCGAA
CACGTGATAGAAGAGCTGTTGGATGAGGACCAGAAAGTTCGGCCCAATGAAGAAAACAATAA
GGACGCAGACTTGTACACGTCCAGGGTGATGCTCAGTAGTCAAGTGCCTTTGGAGCCTCCTC
TTCTCTTTCTGCTGGAGGAATACAAAAATTACCTAGATGCTGCAAACATGTCCATGAGGGTCC
GGCGCCACTCTGACCCTGCCCGCCGAGGGGAGCTGAGCGTGTGTGACAGTATTAGTGAGTG
GGTAACGGCGGCAGACAAAAAGACTGCAGTGGACATGTCGGGCGGGACGGTCACAGTCCTT
GAAAAGGTCCCTGTATCAAAAGGCCAACTGAAGCAATACTTCTACGAGACCAAGTGCAATCC
CATGGGTTACACAAAAGAAGGCTGCAGGGGCATAGACAAAAGGCATTGGAACTCCCAGTGC
CGAACTACCCAGTCGTACGTGCGGGCCCTTACCATGGATAGCAAAAAGAGAATTGGCTGGC
GATTCATAAGGATAGACACTTCTTGTGTATGTACATTGACCATTAAAAGGGGAAGATAG
